# Editorial: Early Intervention in Mood Disorders

**DOI:** 10.3389/fpsyt.2021.799941

**Published:** 2021-12-09

**Authors:** Steven Marwaha, June S. L. Brown, Chris G. Davey

**Affiliations:** ^1^Institute for Mental Health, School of Psychology, University of Birmingham, Birmingham, United Kingdom; ^2^Specialist Mood Disorders Clinic, Birmingham and Solihull Mental Health NHS Foundation Trust, Birmingham, United Kingdom; ^3^Psychology Department, Institute of Psychiatry, Psychology and Neuroscience, King's College London, London, United Kingdom; ^4^Department of Psychiatry, The University of Melbourne, Melbourne, VIC, Australia

**Keywords:** early intervention, mood disorder, depression, bipolar (affective/mood) disorders, school

Mood disorders such as depression and bipolar disorder are a major global mental health challenge. Both are common, with the prevalence of depression being 4.4% (1) and bipolar disorder 2.4% (2). They are highly recurrent disorders, with depression causing the greatest burden of disease in young people aged 10–24, and bipolar disorder the fourth greatest (3). The peak incidence for these conditions is in the 10–24 year age group. Mood disorders damage education, relationships and personal development, and are associated prospectively with physical health problems, and with early death (4, 5). Their financial costs are very large indeed (6) and closely linked to work days lost, presenteeism, and absenteeism (7, 8).

Early intervention, meaning action in prevention and treatment of young people at elevated risk or after first onset of mood disorders is critical to reducing the major morbidity and harms of the conditions. It is desirable and important that the interventions are interdisciplinary in nature as the scale of the challenge means that multi-level approaches are needed. This includes interventions at the population level, schools, community, or in mental health or primary care clinics. These hold the promise of changing trajectory and life course of the large population of people who are impacted by mood disorders.

The collection of papers brought together in this Research Topic series offer an important glimpse into early intervention both now and for the future. Using innovative smart-phone technology to assist in monitoring of mood symptoms and rest-activity data appears critically important to the field. The work of Melbye et al. offers insight into how automatically generated smart-phone data could be linked to symptoms in 40 newly diagnosed patients with BD. Similarly, a review of 30 studies on online interventions that focussed on indicated prevention of mood disorders in people with subthreshold symptoms appears to show promise in clinical outcomes, though engagement rates may be fairly modest, suggesting that human support remains important van Doorn et al. A key task in early intervention is being able to stratify treatment. This relies on staging young people correctly when they seek help from youth mental health services so that needs, and help are matched. Work is underway in how this could be done at a service scale and in an automated fashion, with help from mental health staff as needed Iorfino et al..

New or re-purposed treatments are badly needed for people with mood disorders. Therapies are being developed and specialized for young people with at-risk features of bipolar disorder and Scott and Meyer describe the development and initial piloting of treatment on 14 young people at risk of developing bipolar disorder, focussing on problem-solving, reducing sleep-wake cycle disturbances, and self-management of rumination Scott and Meyer. It is also important to recognize that not all adolescents respond to medication or psychological treatment. There is emerging evidence for a different approach in repetitive transcranial magnetic stimulation (rTMS) in adolescent depression (9). Oberman et al. provide an erudite review in this area Oberman et al..

School based interventions are an important area of early intervention for young people (10). De Jonge-Heesen et al. report that providing a CBT depression prevention program for 130 adolescents with elevated depressive symptoms can lead to reduction in comorbid anxiety, leading to better outcomes in this population De Jonge-Heesen et al. Pile et al. describe a highly innovative approach to a school based intervention for the prevention of depression by reviewing the literature and describing the co-development of an imagery rescripting protocol for 37 young people with depression symptoms Pile et al..

Finally, Lagerberg et al. provide data in a much needed area; that of comorbid substance misuse in people in the early phases of bipolar disorder. They report data from 112 individuals which shows substance abuse decreased in the early phases of bipolar disorder and that stopping alcohol misuse may lead to substantial benefits in the clinical course of the condition.

This collection of research papers indicates that the field of early intervention in mood disorders is beginning to thrive, but there is much work to do, in order to meet the challenge of these disabling conditions.

## Author Contributions

SM wrote the initial draft of the manuscript. JB and CD contributed to content, text, and style iteratively. All authors contributed to the article and approved the submitted version.

## Conflict of Interest

The authors declare that the research was conducted in the absence of any commercial or financial relationships that could be construed as a potential conflict of interest.

## Publisher's Note

All claims expressed in this article are solely those of the authors and do not necessarily represent those of their affiliated organizations, or those of the publisher, the editors and the reviewers. Any product that may be evaluated in this article, or claim that may be made by its manufacturer, is not guaranteed or endorsed by the publisher.

## References

[1] World Health Organisation. Depression and Other Common Mental Disorders: Global Health Estimates. Geneva: World Health Organization (2017).

[2] Merikangas KR Jin R He JP Kessler RC Lee S Sampson NA. Prevalence and correlates of bipolar spectrum disorder in the world mental health survey initiative. Arch General Psychiatry. (2011) 68:241–51. 10.1001/archgenpsychiatry.2011.12 21383262 PMC3486639

[3] Gore FM Bloem PJ Patton GC Ferguson J Joseph V Coffey C. Global burden of disease in young people aged 10–24 years: a systematic analysis. Lancet. (2011) 377:2093–102. 10.1016/S0140-6736(11)60512-6 21652063

[4] Watkins LL Koch GG Sherwood A Blumenthal JA Davidson JR O'Connor C. Association of anxiety and depression with all-cause mortality in individuals with coronary heart disease. J Am Heart Assoc. (2013) 2:e000068. 10.1161/JAHA.112.000068 23537805 PMC3647264

[5] Pinquart M Duberstein P. Depression and cancer mortality: a meta-analysis. Psychol Med. (2010) 40:1797–810. 10.1017/S0033291709992285 20085667 PMC2935927

[6] Simon J Pari AA Wolstenholme J Berger M Goodwin GM Geddes JR. The costs of bipolar disorder in the United Kingdom. Brain Behav. (2021) 11:e2351. 10.1002/brb3.2351 34523820 PMC8553306

[7] Evans-Lacko S Knapp M. Global patterns of workplace productivity for people with depression: absenteeism and presenteeism costs across eight diverse countries. Soc Psychiatry Psychiatric Epidemiol. (2016) 51:1525–37. 10.1007/s00127-016-1278-4 27667656 PMC5101346

[8] Marwaha S Durrani A Singh S. Employment outcomes in people with bipolar disorder: a systematic review. Acta Psychiatrica Scand. (2013) 128:179–93. 10.1111/acps.12087 23379960

[9] Hett D Rogers J Humpston C Marwaha S. Repetitive transcranial magnetic stimulation (rTMS) for the treatment of depression in adolescence: a systematic review. J Affect Disord. (2021) 278:460–9. 10.1016/j.jad.2020.09.058 33011525

[10] Brown JS Blackshaw E Stahl D Fennelly L McKeague L Sclare I. School-based early intervention for anxiety and depression in older adolescents: a feasibility randomised controlled trial of a self-referral stress management workshop programme (“DISCOVER”). J Adolescence. (2019) 71:150–61. 10.1016/j.adolescence.2018.11.009 30738219

